# Bimetallic metal–organic frameworks (BMOFs) for dye removal: a review

**DOI:** 10.1039/d4ra06626j

**Published:** 2024-10-08

**Authors:** Kawan F. Kayani

**Affiliations:** a Department of Chemistry, College of Science, Charmo University Chamchamal Sulaimani 46023 Kurdistan Region Iraq; b Department of Chemistry, College of Science, University of Sulaimani Qlyasan Street Sulaymaniyah Kurdistan Regional Government 46001 Iraq kawan.nasralddin@univsul.edu.iq

## Abstract

Safe drinking water and a clean living environment are essential for good health. However, the extensive and growing use of hazardous chemicals, particularly carcinogenic dyes like methylene blue, methyl orange, rhodamine B, and malachite green, in both domestic and industrial settings, has led to a scarcity of potable water and environmental challenges. This trend poses a serious threat to human society, sustainable global development, and marine ecosystems. Consequently, researchers are exploring more advanced methods beyond traditional wastewater treatment to address the removal or degradation of these toxic dyes. Conventional approaches are often inadequate for effectively removing dyes from industrial wastewater. In this study, we investigated bimetallic metal–organic frameworks (BMOFs) as a solution to these limitations. BMOFs demonstrated outstanding dye removal and degradation capabilities due to their multifunctionality, water stability, large surface area, adjustable pore size, and recyclability. This review provides a comprehensive overview of research on dye removal from wastewater using BMOFs, including their synthesis methods, types of dyes, and processes involved in dye removal, such as degradation and adsorption. Finally, the review discusses the future potential and emerging opportunities for BMOFs in sustainable water treatment.

## Abstract 2

The study on the synthesis and properties of bimetallic metal–organic frameworks (BMOFs) and their applications for dye removal.

## Graphical abstract

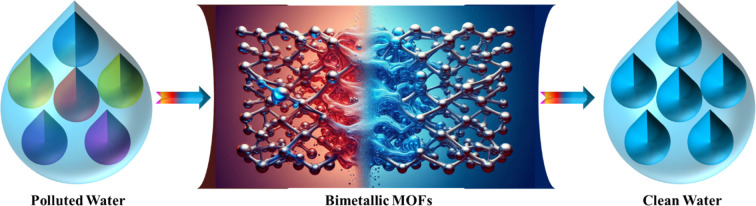

## 1. Introduction

**Fig. 1:**
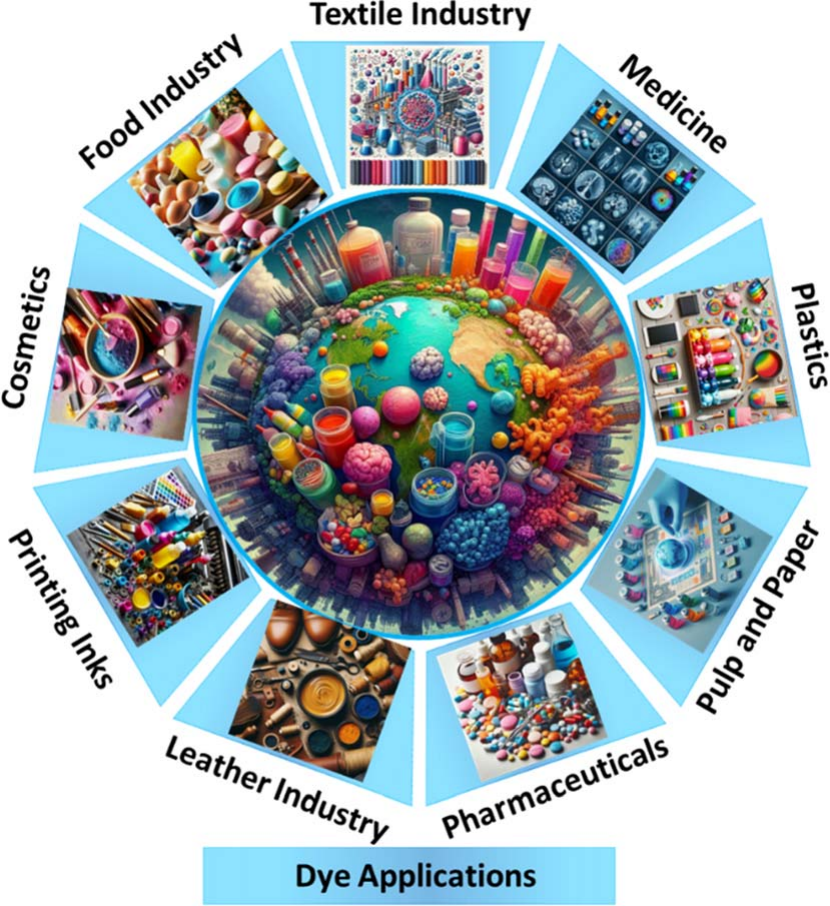
Different types of dyes and their potential industrial applications.

Persistent organic pollutants are commonly found in the waste products of the chemical, dyeing, pharmaceutical, and paper industries, posing a significant environmental pollution challenge. As a result, there is an urgent need for cost-effective and efficient methods to manage and reduce these pollutants.^1,2^ The rapid expansion of the global population, climate change, and industrial progress have significantly impacted water quality, contributing to a growing global freshwater crisis. In this context, various users and polluters of freshwater play a major role in depleting this essential resource.^3–5^ Notably, fabric dyeing is one of the primary contributors to water pollution, with textile dyeing ranking as the second-largest source of water contamination worldwide.^6^ Among the most frequently used dyes, substances like MB,^7^ RhB,^8^ MO,^9^ CR,^10^ MR,^11^ and CV are prominent industrial pollutants originating from diverse sectors such as textiles,^12,13^ cosmetic,^14^ leather,^15^ food,^16^ pharmaceutical,^17^ paint and varnish,^18,19^ and pulp^20^ and paper industries.^21^ As shown in Fig. 1, a recent estimate indicates that approximately 7 × 10^8^ kg of dyes are produced each year. However, due to inefficiencies in the dyeing processes, the textile industry contributes up to 7 × 10^8^ kg of these dyes to wastewater annually during dyeing and finishing operations.^22^ Therefore, it is crucial to remove dyes from wastewater.

Several methods have been employed to remove dye pollutants to date, including ozonation,^23^ filtration,^24^ bioadsorption,^25,26^ biofilm reactors,^27,28^ electrocoagulation,^29^ ion exchange removal,^30,31^ adsorption,^32,33^ catalytic reduction,^34,35^ photocatalytic degradation,^36,37^ and biological/aerobic.^38,39^ The use of various advanced material applications is among the most effective approaches for dye removal. Significant progress in nanomaterials, such as metal oxides,^40–43^ carbon dots,^44–51^ sulfur dots,^52^ and metal–organic framework (MOF)-based nanoparticles,^53–55^ has been notable in the early 21st century. These synthesized materials have proven successful in environmental treatment and protection. Notably, MOF-based materials are the most widely used in this domain due to their cost-effectiveness, diverse configurations and structures, high thermal and mechanical stability, adjustable pore properties, extensive surface area, and reusable metal sites.^56–61^

MOFs have recently attracted considerable attention for their photocatalytic properties. These functional hybrid materials are formed by connecting organic ligands with metal ions, and they offer a variety of advantageous features, including high porosity, large surface area, exposed metal sites, and the flexibility to be customized through various material combinations and synthesis techniques.^62–64^ These qualities make MOFs highly useful in a wide range of applications, including sensing,^65,66^ catalysis,^67–70^ drug delivery,^71–73^ pollutant removal from water,^74,75^ and energy storage or conversion.^76^

BMOFs are created by linking two distinct metal ions with an organic ligand.^77^ Although metal substitutions are commonly used in the chemistry of oxides and intermetallic compounds, this approach has been less frequently applied to MOF production, likely because metallic centers are generally more associated with purely inorganic materials than with hybrid ones.^78^ Nonetheless, bimetallic MOFs have recently gained widespread interest due to their outstanding structural and chemical stability, significant porosity, and potential applications in gas adsorption,^79^ separation,^80^ catalysis,^81^ sensing,^82,83^ and biochemistry.^84^ Thanks to their high porosity and numerous adsorption sites, BMOFs have demonstrated advantages over monometallic MOFs in various applications. They also show promise as precursors or templates for the development of BMOF-derived photocatalysts, which possess more active sites and enhanced conductivity compared to their monometallic counterparts.^85^ As a result, the use of BMOFs for dye removal holds great promise and is highly significant for environmental applications.

**Fig. 2:**
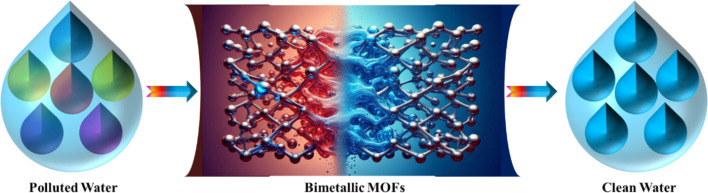
Schematic diagram depicting BMOFs for dye removal.

Several research groups have investigated various methods for addressing dye-contaminated wastewater using different materials.^86–91^ This literature review provides an overview of the use of BMOFs for removing dye pigments from wastewater, emphasizing the effectiveness of BMOFs as adsorbents compared to other materials in dye removal. Additionally, the review explores the mechanisms of BMOF-dye adsorption and the future prospects for using BMOFs in this application. To our knowledge, no existing review paper offers a comprehensive discussion on dye removal specifically with BMOFs. Thus, this review aims to present the latest information on the application of BMOFs for dye removal from aqueous solutions, along with future perspectives, as illustrated in Fig. 2.

### 1.1 Categorization of dyes

**Fig. 3:**
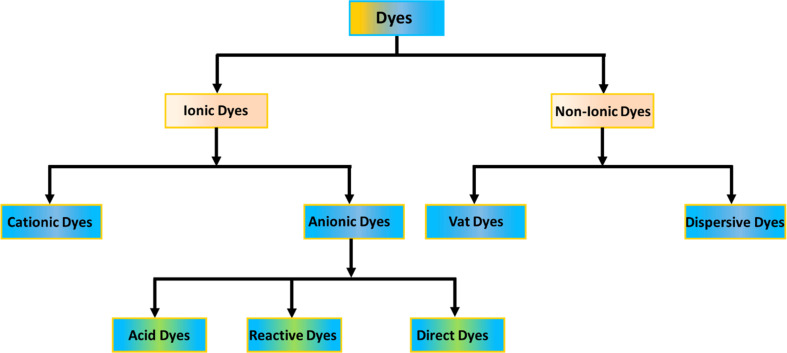
Categorization of dyes.

Dyes are commonly classified based on their structural or functional groups, color, and ionic charge when dissolved in water.^92^ Since the ionic classification of dyes significantly impacts adsorption efficiency, this review adopts that classification. As illustrated in Fig. 3, dyes are divided into ionic and non-ionic categories. Non-ionic dyes include vat and disperse dyes, while ionic dyes are further categorized as cationic (basic) and anionic (direct, acidic, and reactive).^93^

**Fig. 4:**
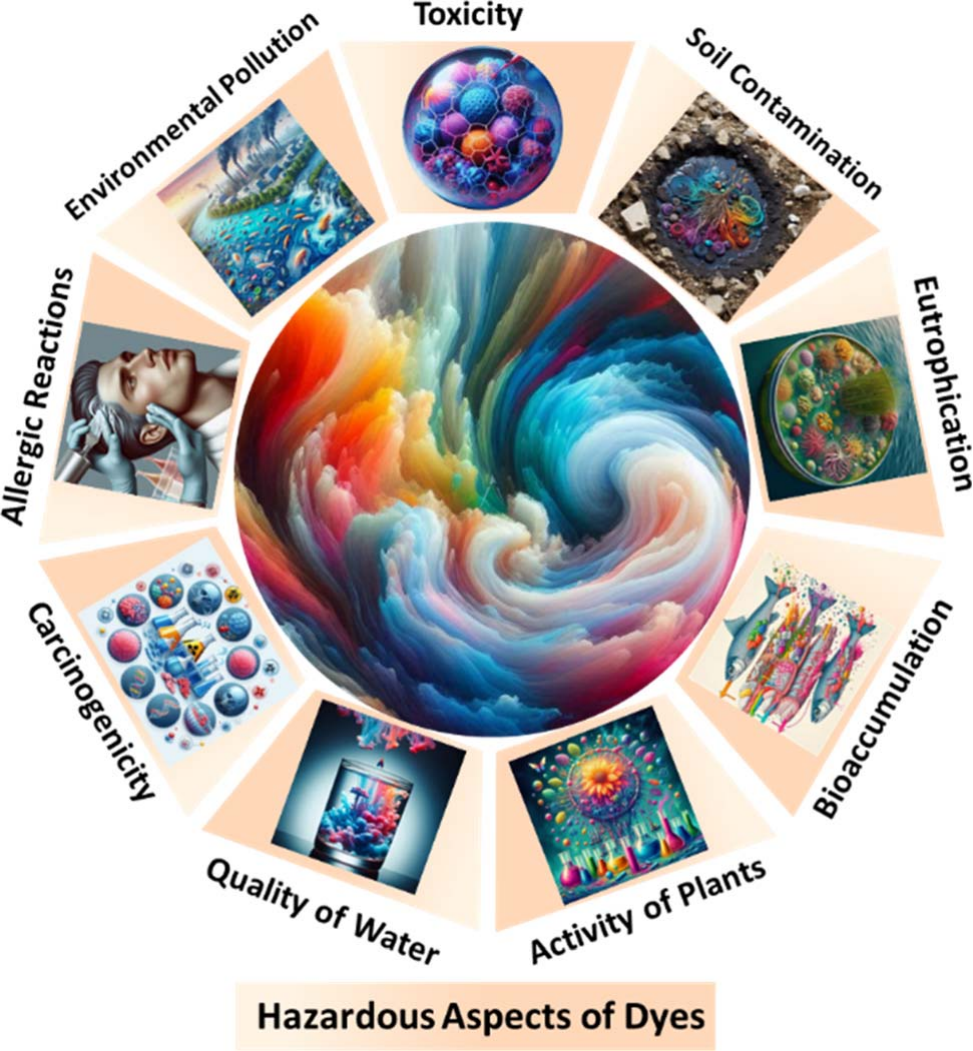
Hazardous aspects of dyes.

The components of dyes make them toxic. Typically, the presence of dyes in water bodies can affect the photosynthesis of aquatic life by blocking sunlight transmission. More concerning is that many dyes are mutagenic, carcinogenic, or teratogenic to both animals and humans. Dye molecules in wastewater are known to cause dysfunction in multiple human organs. Direct, cationic, acidic, and disperse dyes can all contribute to the development of benign and malignant tumors, with direct dyes being linked to bladder cancer. Reactive dyes can cause dermatitis, rhinitis, allergic conjunctivitis, and occupational asthma. Additionally, many of the dyes discussed are carcinogenic, highlighting the importance of effectively treating dye-contaminated wastewater from dye manufacturing plants.^94^ As shown in Fig. 4.

## 2. Mechanism for dye removal

**Fig. 5:**
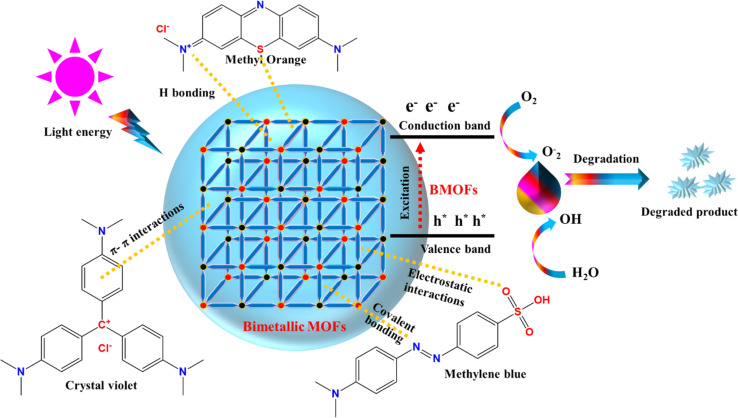
Schematic diagram of dye adsorption and degradation.

The adsorption of molecules on the surface of an adsorbent can occur in two distinct ways, depending on the interaction between the solid surface (BMOFs) and the adsorbed molecules: physical and chemical sorption. In physisorption, electrostatic interactions and van der Waals forces are involved, making the process reversible. A possible mechanism is illustrated in Fig. 5. The adsorption mechanism on the adsorbent surface involves three types of interactions: (1) electrostatic interactions, (2) hydrogen bonding, and (3) π–π stacking interactions, all contributing to the enhanced adsorption of dye molecules. In contrast, chemisorption involves strong covalent bonds as the primary interaction between the adsorbent and adsorbate, leading to diffusion from the surface into the material's interior, typically forming a monolayer.

In recent years, the use of biological sources for dye degradation has emerged as a promising and eco-friendly alternative to traditional chemical methods for removing dyes from polluted water and soil. This approach harnesses natural biological processes, such as microbial activity and enzymatic reactions, which can break down harmful dye compounds without generating toxic byproducts. Alongside biological methods, researchers have focused on developing engineered BMOFs for enhanced dye degradation. These BMOFs are synthesized from various metal combinations and organic linkers, offering a unique structure with high porosity and active sites that facilitate the breakdown of complex dye molecules.

One particularly promising method is the photocatalytic degradation of dyes using BMOFs. Under light irradiation, BMOFs can generate reactive species, such as hydroxyl radicals and superoxide anions, which play a crucial role in oxidizing and decomposing dye molecules. The presence of two different metals in the BMOF structure enhances light absorption and charge separation, making the photocatalytic process more efficient. This process, referred to as dye degradation by BMOFs in the presence of light, is illustrated in Fig. 5, showing how these materials can harness light energy to degrade dyes into less harmful compounds.

## 3. BMOF properties, and synthesis

Although single-metal MOFs offer a broad range of structural compatibilities, using transition metals (Fe, Zn, Co, Ni) in BMOFs can lower costs and improve catalytic performance.^95,96^ BMOFs can be categorized into two types based on their spatial arrangement: different metals in separate secondary building units (SBUs) or different metals within the same SBUs. The latter typically exhibits greater catalytic activity due to its denser structure, improved stability, and enhanced electron transfer.^97^ Combining two metal cations can boost conductivity and promote oxidation reactions between different metal sites within the MOF, leading to higher catalytic efficiency. This method of integrating functional components can create multifunctional complexes with superior properties, enhancing activity for redox reactions, supercapacitors, and other processes.^98^ Although BMOFs are still under development, increasing research highlights their promising potential for various practical applications.

BMOF-based composites offer several benefits compared to monometallic MOFs: (i) they combine different metallic elements, organic ligands, and structures from monometallic MOFs, leading to a wide range of compositions and functions; (ii) they enhance pore development, with their synthesis being relatively simple and gentle; (iii) their structured arrangement of metal ions and ligands improves the fixation, dispersion, stability, and catalytic activity of the materials.^99,100^ Considering these benefits, numerous studies have documented nanomaterials based on BMOFs with diverse compositions and structural properties that are extensively employed in environmental pollution management.^101,102^

### 3.1 Advantages of bimetallic MOFs over monometallic MOFs

Bimetallic MOFs present numerous advantages over their monometallic counterparts, particularly in terms of enhanced stability. The interaction between two distinct metal ions in BMOFs leads to improved structural integrity. As a result, they typically exhibit superior thermal, chemical, and mechanical stability compared to monometallic MOFs, which is crucial for withstanding extreme conditions such as high temperatures, acidic or basic environments, and varying pressures.^99,103,104^ Characterized by their high porosity and abundance of adsorptive sites, BMOFs outperform monometallic MOFs in several applications.^105^ They also hold promise as precursors or templates for developing BMOF-derived photocatalysts, which offer a larger number of active sites and greater conductivity than their monometallic-derived counterparts.^106^

Moreover, the inclusion of two metals allows for more precise tuning of a MOF's properties, such as pore size, electronic structure, and adsorption capabilities. This ability to customize these properties enhances performance.^107^ The synergistic effects between the metals can result in improved redox behavior, better conductivity, and increased adsorption capacity. The dual-metal system also facilitates the adjustment of electronic, magnetic, and optical properties, making BMOFs highly versatile and suitable for a wider range of applications.^108–110^

### 3.2 Synthesis of BMOFs

**Fig. 6:**
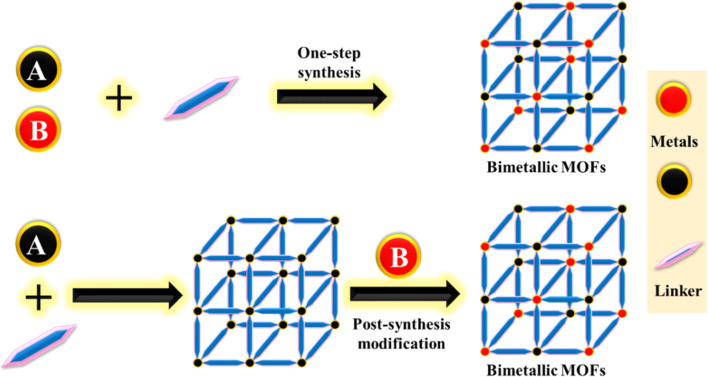
Illustration of synthesis strategies for BMOFs using one-step and post-synthetic approaches.

Various methods have been developed for the synthesis of MOFs, including hydrothermal,^111^ solvothermal,^112^ microwave,^113^ electrochemical,^114^ sonochemical,^115^ and reflux techniques.^116^ Several of these techniques can also be used to synthesize bimetallic MOFs. Typically, synthesis methods fall into two main categories: one-pot synthesis and post-synthetic modification^117^ (Fig. 6). Unlike the synthesis of monometallic MOFs, the synthesis of BMOFs requires strict control of nucleation rates and growth kinetics due to the differing reaction kinetics of the two types of metal ions or clusters.

#### 3.2.1 Direct, and solution based synthesis

BMOFs can be created by combining metal salts using a solvothermal process. However, simply mixing different metal salts does not guarantee the formation of BMOFs.^118^ To avoid the formation of mixed phases and achieve a specific structure, it is important to control factors such as solubility, molar ratios of metal ions, and pH. One promising approach for creating BMOFs with a defined composition involves synthesizing predefined secondary building units (SBUs) as precursors for the metal nodes.^119^

Several bimetallic or bi-ligand MOFs are solid solutions with adjustable ratios of ligands or metals. These BMOFs can be created directly by employing multiple ligands or metals, or through post-synthetic modification. The term “solid solution” is used because these MOFs do not have a completely random arrangement of ligands or metals.^120,121^

#### 3.2.2 One pot synthesis (OPS)

The OPS method, also known as ‘one-pot’ synthesis, has significantly improved the process of integrating secondary metal centers into MOFs for various applications.^122^ BMOFs can be created by mixing both metal salts in the same reaction mixture, leading to structures that contain two metal species. This method simplifies the synthesis process and reduces the need for intermediate steps. MOFs with these additional metal nodes often exhibit complex and delicate networks, enhancing their potential applications due to unique synergistic effects. The success of this synthesis relies on selecting the right precursors, optimizing reaction conditions, and carefully adjusting parameters to produce high-quality BMOFs.^103,123^

#### 3.2.3 Post synthetic modification method (PSM)

Another effective method for fabricating BMOFs is post-synthetic modification (PSM). This approach allows for either the exchange of pre-synthesized metal clusters or organic ligands in a MOF or for altering the MOF structure to introduce secondary metal nodes. The success of PSM depends on the stability, porosity, and crystallinity of the initial structure. PSM mainly involves the exchange of metal ions and the elimination–addition of metals.^103^ One of the main challenges is to precisely control the ratio of the two types of metal sites in BMOFs across a broad range.

The first approach, metal ion exchange, is a highly effective method for synthesizing BMOFs. In this method, monometallic (M) MOFs are typically placed into a solution containing secondary metal ions with similar properties, facilitating ion exchange with the metal nodes in the framework. The degree of exchange can be controlled by adjusting experimental parameters.^124^ In summary, BMOFs can be synthesized *via* ion exchange using two types of metal ions with similar properties.

The second strategy, metal elimination–addition, involves the sequential removal of some metal sites in monometallic MOFs to create vacancies, which are then filled with different metal ions. The new metal ions should have a similar charge and coordination mode to those of the ions being replaced.^125,126^

#### 3.2.4 Template method

A method involving templates can effectively regulate the composition of metal ions in BMOFs. Furthermore, this synthesis approach can be used to create hollow BMOFs, offering benefits such as increased active sites and improved mass transport. There are two main approaches to this synthesis method: the self-template method and the exterior-template method. The self-template method is a straightforward way to produce hollow BMOFs, as there is no need to remove the template; the process involves dissolution-regrowth. In contrast, the exterior-template method utilizes a sacrificial template that must be removed after synthesis.^119^

#### 3.2.5 Core–shell (CS) BMOFs

In core–shell (CS) BMOFs, the outer shell and inner core are constructed using different metal centers. Seed-induced growth has been proven to be an effective method for producing core–shell nanomaterials. Through epitaxial growth, two MOFs with similar lattice parameters can be combined to form CS MOFs.^127^ Another method, post-synthetic selective exchange of metal ions within the framework, can also be utilized to create CS BMOFs. This is possible because the metal sites in the core and near the surface of the MOF exhibit different flexibilities and, consequently, distinct reactivities. Therefore, by carefully controlling the post-synthetic metal exchange process, CS BMOFs can be produced through selective transmetalation.^108^

## 4. Dye removal by BMOFs

Rapid industrial expansion is a significant contributor to water pollution.^128^ Among the various pollutants present in water bodies, organic dyes are particularly problematic, as they pose serious risks to both humans and animals. A substantial portion of industrial wastewater consists of dye-contaminated effluents.^129^ Globally, approximately 800 000 tons of dye are produced each year, with nearly 20% of these effluents being released into the environment regularly without adequate public or environmental awareness.^130^ Therefore, it is crucial to handle these corrosive dyes with care. Numerous porous adsorbents have been investigated for their effectiveness in removing dyes from water.^131–135^ Among these, BMOF-based porous materials exhibit remarkable capabilities for dye removal in aqueous environments.

The following sections will explore recent advancements in BMOFs for the removal of both cationic (C) and anionic (A) dyes from water samples. Dyes can be categorized into three groups: cationic (C), anionic (A), and nonionic, each encompassing numerous types of dyes. Anionic dyes include direct, reactive, and acid dyes, while basic dyes represent cationic dyes, and dispersed dyes represent nonionic dyes.^136^ For greater efficiency, the effect of pH must be considered. The initial pH of the dye solution significantly influences factors such as ionization levels, surface charge density, and the adsorption capacity of the adsorbent, playing a crucial role in the overall adsorption process. The adsorption rate fluctuates with changes in the pH of the medium. At low pH levels, the removal efficiency of cationic dyes decreases, while for anionic dyes, it increases due to the highly protonated surface of the adsorbent, which favors the adsorption of anionic groups and enhances the overall process. However, at high pH levels, the opposite effect is observed.^137^

### 4.1 Removal of cationic (C) dyes

Cationic (C) dyes in water carry a positive charge, are highly soluble, and exhibit strong coloration even at low concentrations. These dyes can be readily and effectively adsorbed onto the negatively charged surfaces of adsorbents through electrostatic attraction. However, the efficiency of removal can vary based on the surface area and the functional groups available on the adsorbent's surface.

#### 4.1.1 Methylene blue removal

Methylene blue (MB) is frequently utilized as a benchmark dye in microfiltration and adsorption research due to its molecular characteristics, which make it suitable for various applications, particularly in the medical field. Nevertheless, if not properly treated before disposal, it can pose environmental risks. Studies have shown that overexposure to MB can lead to methemoglobinemia by directly oxidizing hemoglobin. Additionally, it has the potential to induce issues related to hemolysis, particularly in newborns. Prolonged exposure to methylene blue may ultimately result in significant anemia.^138^ Hence, the hazardous and highly pigmented MB dye needs to be eliminated from wastewater prior to its release into the environment.

**Fig. 7:**
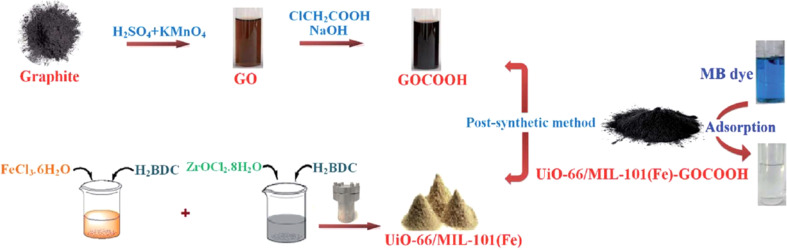
Diagram showing the synthesis of the composite and images illustrating the adsorption process. From ref. 139 with permission. Copyright 2020, Royal Society of Chemistry.

This study by Eltaweil *et al.*^139^ introduces a new composite as an effective adsorbent for cationic MB dye. The UiO-66/MIL-101(Fe) BMOF was created using a solvothermal method. The adsorption capability of the UiO-66/MIL-101(Fe)–GOCOOH composite was evaluated using a batch technique, showing that it had a higher adsorption capacity compared to the BMOF alone. Adsorption isotherms and kinetic studies indicated that MB dye adsorption on the composite follows the Langmuir isotherm model and both pseudo-first-order and pseudo-second-order kinetic models. Additionally, thermodynamic data suggested that the adsorption process is endothermic, spontaneous, and involves both physisorption and chemisorption. The newly developed composite also demonstrated good reusability, making it a highly promising adsorbent for efficiently treating dye-containing industrial effluents. As shown in Fig. 7.

**Fig. 8:**
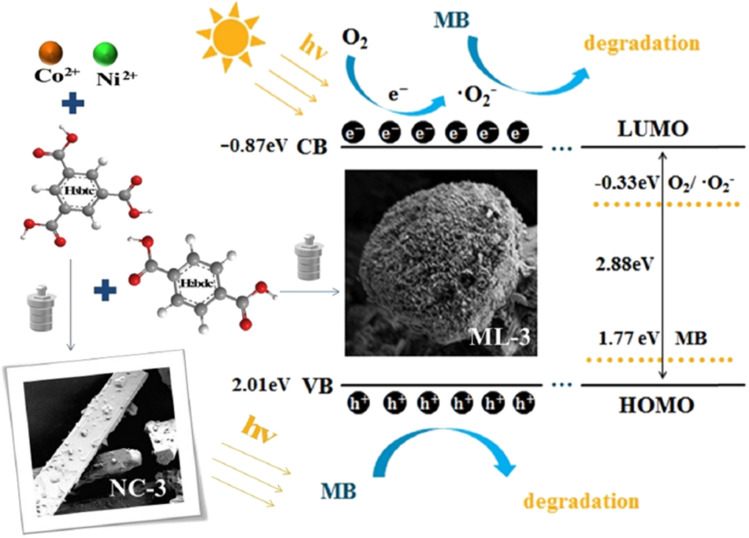
Diagram of sample synthesis and photocatalytic mechanism. Form ref. 140 with permission. Copyright 2022, Elsevier.

Shan *et al.*^140^ synthesized a series of MOF materials using a solvothermal method, with trimellitic acid and terephthalic acid reacting with nickel and cobalt metal salts for the photodegradation of MB under xenon light irradiation. Among these, ML-3, a bimetallic mixed-ligand MOF material with a specific ratio and a tight-flower structure based on the solid block structure NC-3, exhibited outstanding degradation performance against MB. While NC-3 achieved a degradation efficiency of 80.6% in 120 minutes, ML-3 reached 97.8% in the same timeframe. Radical trapping experiments and Mott–Schottky analysis revealed that h^+^ and ˙O_2_^−^ were the primary active substances in the photocatalytic degradation mechanism. The study found that the redox potential of MB (1.77 eV) was lower than the valence band (VB) potential of ML-3 (2.01 eV), allowing h^+^ to directly oxidize MB. Consequently, the materials synthesized in this study are effective for wastewater treatment and show significant potential for environmental applications, as shown in Fig. 8.

Ma *et al.*^141^ synthesized Ce/Co BMOFs. The photocatalytic activity was tested under Xe lamp irradiation with MB as a model pollutant. Among the samples, DT-3, with a Ce/Co ratio of 4 : 1 and an H_2_bdc/H_3_btc ratio of 1 : 2, demonstrated the highest photocatalytic performance, achieving a 97.6% degradation efficiency of MB within 120 minutes. Analysis revealed that DT-3 had a bandgap energy of 3.20 eV, with a conduction band potential of −0.75 eV, which is more negative than the standard redox potential of O_2_/˙O_2_^−^, and a VB potential of 2.45 eV, exceeding the redox potential of MB. Overall, the results indicate that the synthesized bimetallic photocatalyst with mixed ligands is effective in degrading the MB dye in wastewater. Table 1 summarizes studies that utilized BMOFs for the removal of MB.

**Table 1:** Degradation of MB by BMOFs

Catalysts	Method	Catalyst dosage	Initial concentration	Temperature °C	pH	Performance%	Ref.
Zn/Co MOFs	Electrosorption	—	2847 mg g^−1^	—	—	90	142
Zn/Co MOFs	Degradation	0.01 g L^−1^	128 mg L^−1^	—	—	40	143
Fe/CoMIL-88B	Degradation	0.8 g L^−1^	0.1 mM	70	10	100	144
Al/Cu MOFs	Adsorption	0.1 g L^−1^	381.68 mg g^−1^	25	4–6	84	145
Zn/Mn MOFs	Degradation	1 g L^−1^	10 mg L^−1^	90	—	91.23	146
Ni/Co MOF@MAC	Adsorption	0.2 g	40 mg L^−1^	25	6	—	147
Co/Fe MOFs	Adsorption	1 g L^−1^	1 mg L^−1^	90	4	90	53
Ni/Zn MOFs	Degradation	40 mg	10 mg L^−1^	—	—	97.4	148
Cu/Co MOFs	Degradation	0.6 g L^−1^	—	45	6.2	93.29	149
Cu/Co MOFs	Degradation	50 mg L^−1^	0.2 mM	150	7.15	100	150
Fe/Cu MOFs	Degradation	0.6 g L^−1^	0.2 mM	25	9.05	100	151
Co/Ni MOFs	Degradation	0.16 g	10 mg L^−1^	—	5	99	152
Cu/Zn MOFs	Adsorption	5 g L^−1^	200 mg L^−1^	25	7	98	153
Ti/Zr MOFs	Degradation	10 mg	20 mg L^−1^	—	—	93.2	154
Co/Ni MOFs	Degradation	150 mg L^−1^	20 mg L^−1^	20	6.23	99.4	155
Co/Ni-MOFs@BiOI	Degradation	0.3 g L^−1^	20 mg L^−1^	—	5	81.3	156
Ni/Co MOFs	Degradation	8 mg	10 mg L^−1^	—	—	100	58
Zn/Co ZIFs	Degradation	1 mg	10 mg L^−1^	—	Neutral	45	157
Fe/Co MOFs	Degradation	20 mg	20 mg L^−1^	—	—	—	158
Tb/Eu MOFs	Adsorption	20 mg	5 × 10^−5^ M	—	—	99	159

#### 4.1.2 Rhodamine B (RhB) removal

RhB is a water-soluble organic dye commonly used to color wool, cotton, silk, paper, and fabrics.^160^ However, RhB contamination in water can be toxic to plants and carcinogenic to living organisms due to its aromatic structures.^161^ Therefore, removing RhB from industrial wastewater before it is released into the environment is essential.

RhB dyes were effectively removed using a new bimetallic Co/Fe-MOF developed by Hu *et al.*^162^ This innovative MOF exhibits photocatalytic activity for degrading RhB. With a catalyst dosage of 200 mg L^−1^, a degradation efficiency of 99.7% was achieved within 30 minutes at a temperature of 25 °C under visible light irradiation.

**Fig. 9:**
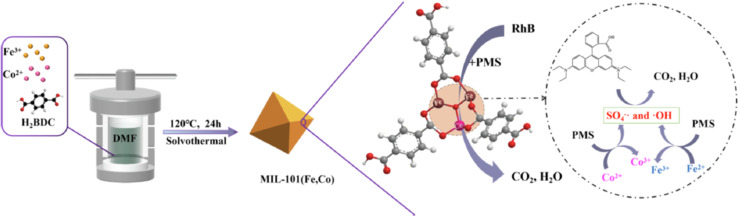
The schematic shows the synthesis of MIL-101(Fe,Co) and its dye degradation mechanism. From ref. 163 with permission. Copyright 2023, Elsevier.

Xiao *et al.*^163^ synthesized cobalt-doped MIL-101(Fe) to enhance the catalytic performance of MIL-101. By optimizing the cobalt doping ratio, MIL-101(Fe,Co) was identified as having the highest catalytic activity. The activation performance of BMOFs for peroxymonosulfate (PMS) was tested using RhB as a model pollutant under the following conditions: [RhB] = 10 mg L^−1^, [catalyst] = 0.2 g L^−1^, and [PMS] = 0.4 g L^−1^, with a reaction time of 15 minutes. The results demonstrated that more than 99% of RhB was degraded within 15 minutes, and the catalyst maintained effective degradation across a broad pH range of 3–11. Additionally, MIL-101(Fe,Co) exhibited excellent stability, retaining over 90% degradation efficiency even after five cycles. As shown in Fig. 9.

A new Cd/Zr-MOF was successfully synthesized. The study examined how the Cd/Zr molar ratio and temperature influence the structure of the Cd/Zr-MOF and its photocatalytic efficiency in degrading RhB under simulated sunlight. The findings revealed that the Cd/Zr-MOF with a 5 : 1 Cd/Zr molar ratio, synthesized at 160 °C, demonstrated the highest photocatalytic performance, achieving a 95.82% degradation efficiency after 105 minutes of irradiation, significantly surpassing the efficiency of pure Cd-MOF.^164^

Table 2 summarizes studies that utilized BMOFs for the removal of RhB.

**Table 2:** Degradation of RhB by BMOFs

Catalysts	Method	Catalyst dosage	Initial concentration	Temperature °C	pH	Performance%	Ref.
Fe/Al MOFs	Degradation	0.10 g/100 mL	10^−4^ M	—	—	99.61	165
Zn/Ru MOFs	Reduction	2 mg mL^−1^	5.0 mM	—	—	99	166
La/Fe MOFs	Degradation	1 g L^−1^	10 mg L^−1^	—	—	96	167
Fe/Co MOFs	Degradation	0.3 g L^−1^	20 mg L^−1^	—	4.7	98.7	168
Co/Fe MIL88/MCC	Degradation	60 mg L^−1^	5 mg L^−1^	—	5.5	87	169
Fe/Ni MOFs	Degradation	5 mg	20 mg L^−1^	—	7	100	170
Fe/Cu MOFs	Degradation	1.19 g L^−1^	10 mg L^−1^	—	Neutral	80.92	171
Bi/Zn MOFs	Degradation	0.5 g L^−1^	10 mg L^−1^	—	—	99	172
Ni/Co-MOF@GNS	Degradation	0.05 mg mL^−1^	25 mg L^−1^	—	11	94	173
Cu/Fe MOFs	Degradation	0.25 g L^−1^	10 mg L^−1^		Neutral	92	174
M/Fe MOFs	Degradation	5 mg	3 10^−5^ M	—	—	85–92	175
M/Fe MOFs	Degradation	9 mg	3 10^−5^ mol L^−1^	—	5	96	176

#### 4.1.3 Other cationic dye

##### Crystal violet (CV)

The CV dye is a vibrant triphenylmethane dye commonly utilized in the textile, dyeing, leather production, printing, and food sectors. It poses health risks, as it is carcinogenic, teratogenic, and mutagenic, even in minimal concentrations.^177^ Jais *et al.* introduced a new material, NiFe-MOF@AHC, designed to effectively remove large-sized pollutants, such as CV dye. The composite showed rapid removal of CV, primarily through chemisorption. The maximum adsorption removal (*Q*_max_) of the composite for CV dye (395.9 mg g^−1^) was 1.5 times lower than that of AHC. Regeneration studies indicated that the removal efficiency decreased after the first cycle but remained consistent until the fourth cycle, suggesting that the solvothermal growth of NiFe-MOF on AHC successfully produced a stable and reusable adsorbent.^178^

##### Methyl violet (MV)

Methyl violet (MV) holds significant importance due to its wide-ranging applications in textiles, paints, and printing inks. It is commonly used for dyeing materials.^179^ In the biomedical field, MV serves as the active ingredient in Gram's stain, which is used for bacterial classification. Additionally, it can be employed as a moderate-level disinfectant, although it has been found to be toxic to most animals. Inhalation of MV may irritate the respiratory tract, while ingestion typically leads to gastrointestinal irritation.^180^ Prolonged or repeated exposure to methyl violet (2B) may cause damage to specific organs.^181,182^ Hence, it is crucial to eliminate this dye from wastewater before releasing it into water bodies.

Thu and colleagues developed BMOFs (FeZn-ZIFs) that were utilized as a heterogeneous catalyst to remove methyl violet 2B dye from an aqueous solution. Under the catalytic conditions of a catalyst dosage of 0.3 g L^−1^, an initial dye concentration of 20 mg L^−1^, and at room temperature, the FeZn-ZIFs demonstrated a 95% removal efficiency of MV.^183^

### 4.2 Anionic (A) dyes

A dyes rely on a negative ion. This category encompasses various dye compounds from different classes, including azoic, anthraquinone, triphenylmethane, and nitro dyes. Despite their structural differences, these dyes share water-solubilizing ionic substituents. Direct dyes are also part of the A dye category, with a significant portion of reactive dyes falling under the group of A azo dyes from a chemical perspective.^137^

#### 4.2.1 Methyl orange (MO)

MO is classified as an A dye that finds widespread application as a pH indicator and is also utilized in industries related to paper and dyeing.^184^ Like other dyes, methyl orange is considered toxic and potentially carcinogenic.^185^ Contact with this dye can lead to symptoms such as diarrhea and vomiting.^186^ Researchers have explored the use of various agricultural waste materials for the removal of methyl orange dye.

Tang *et al.* introduced a series of low-crystalline Fe/Ce-MOFs synthesized using DBD plasma technology as promising photocatalysts. These materials demonstrated outstanding photocatalytic performance, achieving a 93% degradation rate of MO (20 mg L^−1^) within 30 minutes under visible light. This cost-effective and straightforward approach offers potential advantages for the development of other low-crystalline BMOFs with superior performance.^187^

A different study assessed the effectiveness of the Mn/Zn@ZIF-8 nanocomposite in adsorbing MO dye from water. The adsorption isotherm analysis indicated that MO adsorption on Mn@ZIF-8 follows a monolayer pattern, aligning with the Langmuir isotherm. The Mn@ZIF-8 nanocomposite achieved a remarkable *q*_max_ of 406 mg g^−1^, which is notably higher than that of pure ZIF-8. Furthermore, the synthesized Mn@ZIF-8 material demonstrated strong reusability, maintaining up to 92% of its adsorption efficiency after four cycles compared to the initial cycle. Overall, the Mn/Zn@ZIF-8 nanocomposite is a promising candidate for treating industrial wastewater contaminated with MO.^188^

Finally, ZIF-67 and Ni-doped ZIF-67 were selected for their high stability in aqueous environments, porosity, and ease of synthesis. A porous Ni-doped ZIF-67 nanocomposite was created by incorporating nickel (Ni) into the ZIF-67 structure. The resulting Ni@ZIF-67 exhibited excellent adsorption efficiency for removing MO from water. The adsorption performance of Ni-doped ZIF-67 was evaluated under different pH levels, contact times, and dye concentrations. The results showed that Ni@ZIF-67 adsorbed more dye in mildly acidic conditions (*q*_e_ = 24.24 mg g^−1^ at pH 6) compared to acidic (*q*_e_ = 17.69 mg g^−1^ at pH 2) and basic conditions (*q*_e_ = 15.74 mg g^−1^ at pH 10). The adsorption data fit best with the Langmuir isotherm model, indicating a monolayer adsorption process. The maximum adsorption capacity achieved was 151.74 mg g^−1^, with excellent recyclability up to the fifth cycle. Additionally, within a 180 minute contact time, ZIF-67 and Ni-doped ZIF-67 nanocomposites adsorbed 68.5% and 82.9% of MO, respectively.^189^

#### 4.2.2 Congo red (CR)

CR is used in industries such as solar cells, pharmaceuticals, textiles, plastics, and papermaking. CR contains six aromatic rings and two azo functional groups, contributing to its toxicity. It can contaminate water, posing serious risks to humans and marine life, including plants and aquatic organisms. In humans, exposure to CR can lead to toxicity and health issues, such as mutations and respiratory problems. The contamination of water by CR is a significant environmental concern.^190^

**Fig. 10:**
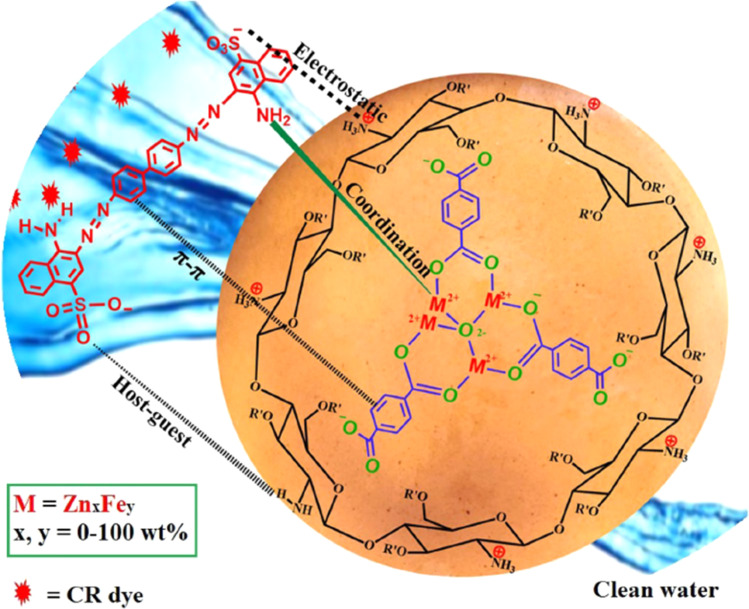
Possible mechanism for CR. From ref. 191 with permission. Copyright 2024, Springer.

Abd El-Monaem *et al.*^191^ developed a bimetallic MOF composite film, Fe/ZnMOF-@CTS, for the removal of CR using an adsorption method. Remarkably, the *Q*_max_ of CR on Fe/MOF-5@CTS reached 219.78 mg g^−1^. In addition, the composite film retained 81.46% of its capacity after more than nine cycles. The selectivity tests revealed that the positively charged composite film exhibited higher selectivity for CR compared to C dyes. Based on practical experiments and analysis, the adsorption mechanism of CR on Fe/MOF-5@CTS is thought to involve electrostatic interactions, host–guest interactions, π–π interactions, and coordination bonds, as illustrated in Fig. 10.

Liu *et al.*^192^ synthesized a bimetallic CoFe-MOF and tested its effectiveness in removing CR from aqueous solutions, demonstrating its potential for treating wastewater containing organic dyes. The CoFe-MOF exhibited an impressive adsorption capacity of 1935.68 mg g^−1^ for CR, significantly higher than the capacities of monometallic Fe and Co MOFs, which were 775.19 mg g^−1^ and 628.93 mg g^−1^, respectively. This suggests that the CoFe-MOF has more defects, leading to enhanced adsorption efficiency. The results indicate that the synthesized MOF materials could be promising candidates for treating organic dye pollution, particularly A dyes, in wastewater.

Table 3 summarizes studies that utilized BMOFs for the removal of CR.

**Table 3:** Degradation of CR by BMOFs

Catalysts	Method	Catalyst dosage	Initial concentration	Temperature °C	pH	Performance%	Ref.
Lac@Co/Cu MOFs	Adsorption	10 mg	100 mg L^−1^	40	7	95	193
Ni/Co MOFs	Adsorption	0.6 g L^−1^	0.4 g L^−1^		4	85	194
Fe/Al MOFs	Adsorption	0.167 mg mL^−1^	500 mg mL^−1^	25	5	96.7	195
ZIF Zn/Co	Degradation	20.0 mg	5 mg L^−1^	—	—	100	196
Ni/Zn MOFs	Adsorption	20 mg	200 mg L^−1^	30	3	—	197
Co/Cu MOFs	Degradation	—	100 mg L^−1^	50	3.5	95	198

Additionally, another type of dye is summarized in Table 4.

**Table 4:** Other anionic dye removal

Catalysts	Dye	Method	Catalyst dosage	Initial concentration	Temperature °C	pH	Performance%	Ref.
Cu/Co MOFs	Orange G	Degradation	50 mg L^−1^	0.2 mM	25	Neutral	96.4	199
NH_2_–MIL-101(Fe/Co)	Orange G	Degradation	50 mg L^−1^	0.2 mM	25	7	100	199
Fe/Ni MOFs	MO	Degradation	—	10 mg L^−1^	—	7	99	200
Ag/Zn MOFs	Reactive yellow 145	Degradation	—	0.4 g L^−1^	—	—	100	201
La/Ag-MOFs	Sunset yellow	Adsorption	0.02 g	0.02 g	—	4	—	202
Cu/Sn MOFs	Acid-blue 92	Degradation	0.03 g	7 × 10^−5^ M	30	—	86.9	203
La/Sn@MOF	Tartrazine	Adsorption	0.02 g	1.06 × 10^−3^ mmol L^−1^	25	6.41	98.3	204
Ag/Cu-MOFs PES	Reactive black 5 and reactive red 120	Rejections	—	—	—	—	96.4, 98.4	205
Cu/Zn ZIFs	RG, RB and CR	Degradation	—	50 mg L^−1^	—	—	68.3%	206
Fe/Ni MIL-88	Eosin-Y	Degradation	5 mg	20 mg	—	—	—	207
Cu/Fe MOFs	Eosin-Y	Adsorption	0.005 g	4 mg L^−1^	—	—	—	208
Mn/Al MOFs	EBT	Degradation	5.0 mg	10 mg L^−1^	—	—	84.9–100	209
Fe/Ti MOFs	Orange II	Degradation	100 mg L^−1^	50 mg L^−1^	25	5	100	210
Cu/Co ZIFs	Acid orange II	Degradation	5 mg	100 mg L^−1^	25	7	95.3	211
Zn/Co ZIFs	Acid violet 7	Reduction	0.2 g L^−1^	20 mg L^−1^	25	3.7	—	212
GCN/M–FeBTC	RR-195	Degradation	0.5 g L^−1^	0.6 g L^−1^	—	9	99.37	213
Ni/Co MOFs	Reactive red	Adsorption	—	100 mg L^−1^	25	—	—	214

### 4.3 Multiple dye removal

BMOFs feature two distinct metal ions within their structure, which can significantly enhance their properties compared to monometallic MOFs. These improvements often include greater stability, larger surface areas, and superior adsorption or catalytic activities. As a result, BMOFs are highly effective for a range of applications, including dye removal. Table 5 summarizes studies that utilized BMOFs for the removal of multiple dyes.

**Table 5:** Degradation of multiple dye by BMOFs

Catalysts	Dyes	Method	Catalyst dosage	Initial concentration	pH	Performance%	Ref.
Cu/Ni–BTC@SiO_2_	MO, MB	Degradation	10 mg L^−1^	20 mg L^−1^	7	98, 71	215
			10 mg L^−1^				
Zr/Cu MOFs	RhB, MB	Degradation	—	—	7	96, 96	216
Co/Zn MOFs	MB, MG RhB, MV-2B, CR	Reduction	20 mg	0.04 mM, 0.0125, 0.0125, 0.038 and 0.02 mM	—	—	217
Zn/Co MOFs	MB, RR	Adsorption	—	—	—	92, 91	217
Cu/Zr MOFs	MO, MB	Adsorption	—	—	—	—	218
Fe/Ni MOFs	MB, MO	Adsorption	10 mg	20 mg L^−1^	6.5, 6.9	84.8	219
Ni/Zn MOFs	MG, CR	Adsorption	0.25 g L^−1^	20 mg L^−1^	—	—	220
Fe/Cu MOFs	MO, MB	Reduction	1 mg	0.05 mM	—	52, 66	221
Co/Ni MOFs	MB, CR, NR	Adsorption	—	—	5–10	98.34, 93.95, 94.42	222
Co/Fe MOFs	MB, MO	Adsorption	0.01 g L^−1^	200 mg L^−1^	10, 4	70, 81	223
Co/Ni MOFs	AB92, MO, and MB	Adsorption	—	20 mg L^−1^	5	—	224
Zn/Cu MOFs	MG	Degradation	—	10 mg L^−1^	4	89.7	225

## 5. Conclusions and prospects

A new material that functions as both an adsorbent and catalyst, with a high capacity for removing dyes, is eagerly anticipated. Recently, porous structured materials based on BMOFs have demonstrated potential in removing toxic dyes. This review focuses on the removal of both cationic (C) and anionic (A) dyes by various BMOFs. Based on the literature, it can be concluded that BMOFs can serve as superior adsorbents for removing A and C dyes from water compared to other nanomaterials, such as carbon dots (CDs), MOFs, metal oxides, and other materials, due to their higher surface area, pore geometries, ease of functionalization, and the presence of two metal nodes. BMOFs can also be useful in real-time applications.

The review highlights the importance of developing low-cost synthesis methods with minimal time requirements and suggests that more effort be put into real sample analysis using BMOFs. It stresses the need to consider the disposal of spent BMOFs and unremoved dyes after adsorption and degradation. Additionally, it outlines several challenges to be overcome, such as production and regeneration expenses, synthesis methods, stability, and practical real-time applications.

The potential of BMOFs as adsorbents or catalysts in water treatment is emphasized, with a recommendation for research focused on developing smart and straightforward BMOFs through green synthesis methods. Key areas of focus include large-scale production, achieving ultra-high surface area, increasing active sites, enhancing stability and selectivity, enabling ultra-fast removal rates, maximizing adsorption capacity, improving reusability, and facilitating real-time applications. The review suggests the possibility of synthesizing BMOFs using a simple, low-cost method and highlights the crucial role of environmental researchers in advancing research and development in this field.

## Data availability

Data sharing is not applicable to this article as no datasets were generated or analysed during the current study.

## Conflicts of interest

There are no conflicts to declare.
